# Reactive oxygen species-related genes participate in resistance to cucumber green mottle mosaic virus infection regulated by boron in *Nicotiana benthamiana* and watermelon

**DOI:** 10.3389/fpls.2022.1027404

**Published:** 2022-11-11

**Authors:** Huiyan Guo, Xinyue Bi, Zhiping Wang, Dong Jiang, Ming Cai, Mengnan An, Zihao Xia, Yuanhua Wu

**Affiliations:** ^1^ Liaoning Key Laboratory of Plant Pathology, College of Plant Protection, Shenyang Agricultural University, Shenyang, China; ^2^ Green Agricultural Technology Center of Liaoning Province, Shenyang, China

**Keywords:** cucumber green mottle mosaic virus (CGMMV), reactive oxygen species (ROS), boron, *Nicotiana benthamiana*, transcriptome, watermelon, virus-induced gene silencing

## Abstract

Cucumber green mottle mosaic virus (CGMMV) infection causes acidification and rot of watermelon flesh, resulting in serious economic losses. It is widely reported the interaction relationship between boron and reactive oxygen species (ROS) in regulating normal growth and disease resistance in plants. Our previous results demonstrated that exogenous boron could improve watermelon resistance to CGMMV infection. However, the roles of ROS-related genes regulated by boron in resistance to CGMMV infection are unclear. Here, we demonstrated that CGMMV symptoms were alleviated, and viral accumulations were decreased by boron application in *Nicotiana benthamiana*, indicating that boron contributed to inhibiting CGMMV infection. Meanwhile, we found that a number of differentially expressed genes (DEGs) associated with inositol biosynthesis, ethylene synthesis, Ca^2+^ signaling transduction and ROS scavenging system were up-regulated, while many DEGs involved in ABA catabolism, GA signal transduction and ascorbic acid metabolism were down-regulated by boron application under CGMMV infection. Additionally, we individually silenced nine ROS-related genes to explore their anti-CGMMV roles using a tobacco rattle virus (TRV) vector. The results showed that *NbCat1*, *NbGME1*, *NbGGP* and *NbPrx Q* were required for CGMMV infection, while *NbGST* and *NbIPS* played roles in resistance to CGMMV infection. The similar results were obtained in watermelon by silencing of *ClCat*, *ClPrx* or *ClGST* expression using a pV190 vector. This study proposed a new strategy for improving plant resistance to CGMMV infection by boron-regulated ROS pathway and provided several target genes for watermelon disease resistance breeding.

## Introduction

Cucumber green mottle mosaic virus (CGMMV) belongs to the genus *Tobamovirus* in the family *Virgaviridae* (Antignus et al., 1990). It mainly infects watermelon, cucumber, melon, pumpkin and other cucurbit crops (Dombrovsky et al., 2017). Among them, watermelon leaves show chlorotic mottling, the stems turn brown and necrosis, and pulp become rot and discoloration accompanied by sour smell after CGMMV infection, causing serious losses in production (Reingold et al., 1935). CGMMV was firstly reported in Britain in 1935 (Ainsworth, 1935), and has become epidemic in many regions of Europe, Asia, Africa, North America and Oceania (Dombrovsky et al., 2017). CGMMV was reported to infect watermelon in Liaoning province of China in 2006 (Chen et al., 2006), and has been listed as a key quarantine plant virus in 2007 (Chen et al., 2008). CGMMV is transmitted mainly by contact and contaminated seeds (Dombrovsky et al., 2017). Recent studies have shown that *Apis mellifera* and *Myzus persicae* also can transmit CGMMV by carrying pollen and physically through mouth needles (Darzi et al., 2018; Qi et al., 2021). The use of disinfectants based on seed treatments and establishment of rapid detection technology can inhibit the spread of CGMMV to some extent (Sui et al., 2018; Jiao et al., 2019; Torre et al., 2020; Chanda et al., 2021). In recent years, some studies have utilized a variety of omics to identify genes, microRNAs, proteins and important post-transcriptional regulations, such as N6-methyladenosine (m6A) methylation, in response to CGMMV infection in watermelon fruits and leaves (Li et al., 2017; Sun et al., 2017; Sun et al., 2019; Li et al., 2020; He et al., 2021). However, the lack of resistant varieties makes the control of CGMMV on cucurbit crops still challenging (Sugiyama et al., 2006).

As a trace element, boron is essential for plant growth and development (Hu and Brown, 1994). Boron is closely related to the composition of plant cell wall and affects the homeostasis of cell wall and cell membrane (Koshiba et al., 2009). Lack of boron will lead to slower growth and malnutrition of plant organs, while excessive boron will cause toxicity to plants, change the metabolism of glucose, and disturb the photosynthesis of plants (Abboud et al., 2020). Boron can affect the absorption and transportation of other essential elements in plants as well as inhibit some toxic elements. The moderate amount of boron can promote the transport of phosphorus to the root, and accelerate the absorption of zinc to promote flowering (Kumar et al., 2019; Zhao et al., 2020). Boron can reduce the invasion of cadmium into cells and alleviate cadmium poisoning by increasing the cellulose in cell wall (Qin et al., 2020; Wu et al., 2020). In addition, boron can affect phytohormone metabolism to regulate plant growth. It was found that the concentrations of abscisic acid (ABA) and indole-3-acetic acid (IAA) were decreased with the increase of boron concentrations in *Brassica napus* seedlings, while cytokinin and gibberellin biosyntheses were significantly increased with boron supply (Eggert and von Wirén, 2017). The regulation effect of brassinosteroids (BRs) on plant root growth was related to its concentration (Mussiget et al., 2003). The down-regulated expression of *BR6ox1* and *BR6ox2* in boron-deficient plants decreased the concentrations of BRs and inhibited root growth (Zhang et al., 2021). Transcription factors (TFs) are closely related to boron homeostasis in plants. BnaA9. WRKY47 enhances *Brassica napus* adaptation to low boron by activating the boric acid channel gene *BnaA3.NIP5;1* (Feng et al., 2020). The expressions of *AtMYB13* and *AtMYB68* in wild-type yeast increased boric acid tolerance (Nozawa et al., 2006). The signal transduction pathway of plant stress tolerance was regulated by various hormones and TFs (Betsuyaku et al., 2018). Previous studies have shown that CGMMV infection mainly affects the biosyntheses of auxin, gibberellin and ABA, and the expressions of some TFs, such as WRKYs, MYBs and ethylene responsive element binding factor (ERFs) families in watermelon fruits (Li et al., 2017). Similar results were obtained from transcriptome sequencing analysis of CGMMV-infected watermelon leaves (Sun et al., 2019).

Several studies have shown a significant link between boron and plant diseases. Grapevine Pinot gris virus (GPGV) infection can disturb boron content in grapes (Buoso et al., 2020). Combined application of boron and iron can reduce *Fusarium* wilt of banana by increasing mannitol content (Dong et al., 2016). It has been reported that boron affected plant biological activities and stress tolerance by regulating homeostasis of reactive oxygen species (ROS). Boron can improve vanadium tolerance of watermelon by enhancing the expressions of superoxide dismutase (SOD) and catalase (CAT) related genes (Shireen et al., 2021). Combined application of boron and zinc promoted the activity of CAT, peroxidase (POD), polyphenol oxidase (PPO) and phenylalanine ammonia lyase (PAL), which is significant for tomato resistance to early blight disease (Awan et al., 2019). Boron not only regulates the activity of reactive oxygen scavenging enzymes of host itself, but also can enhance membrane lipid peroxidation by destroying the reactive oxygen metabolism system of pathogenic bacteria to inhibit their growth (Huang et al., 2008). Therefore, it is well worth studying the roles of boron in improving plant disease resistance.

Plants regulate ROS homeostasis mainly through enzyme-induced and non-enzyme-induced systems (Mittler et al., 2022). The enzymatic system is involved in CAT, POD, glutathione *S*-transferase (GST) and ascorbate peroxidase (APX), while the non-enzymatic system includes flavonoid metabolism, ascorbic acid metabolism, glutathione metabolism and tocopherol metabolism (Kadota et al., 2015). ROS are produced mainly from POD in cell wall and NADPH oxidase in cell membrane in plants (Kawano, 2003). Excessive ROS will cause oxidative damage, affect the normal function of organs, and even lead to cell death (Halliwell, 2006). However, ROS have positive effects on plants, such as promoting plant growth, cell proliferation and differentiation, and shortening the dormant period (Schopfer, 2002; Oracz et al., 2007; Tsukagoshi et al., 2010). In addition, as signaling molecules, ROS can regulate a variety of TFs, Ca^2+^ signals and hormone signals, which can induce hypersensitive response (HR) and expressions of other resistance genes (Yoshioka et al., 2009). Many enzymes that involved in ROS metabolism are essential for antiviral process in plants. For example, maize chlorotic mottle virus (MCMV) P31 hijacks *ZmCat1* to promote viral accumulation by attenuating catalase activity and salicylic acid (SA)-mediated defense response (Jiao et al., 2021). Bamboo mosaic virus (BaMV) infection induces upregulation of *NbGSTU4* expression in *N. benthamiana* to eliminate oxidative stress, while *NbGSTU4* binds to the 3′ untranslated region (UTR) of BaMV RNA in a glutathione-dependent manner to enhance virus replication (Chen et al., 2013). The promoters of class III peroxidase (*OsPrx*) genes bind with transcription factor BZR1 of brassinosteroid (BR) pathway directly to suppress ROS burst in rice, which regulates BR-mediated susceptibility to rice black-streaked dwarf virus (RBSDV) (Zhang et al., 2019).

CGMMV infection affects energy metabolism, hormone transduction, and even destroys cell structure in watermelon (Li et al., 2020). Previous experiments in our laboratory showed that spraying boron improved watermelon resistance to CGMMV infection (Bi et al., 2022). In this study, we further confirmed that spraying boron could reduce CGMMV accumulations and alleviate viral symptoms in *N. benthamiana*. We demonstrated that ROS-related genes regulated by boron participated in resistance to CGMMV infection in *N. benthamiana* and watermelon by RNA sequencing (RNA-seq) analyses and virus-induced gene silencing (VIGS) assays, respectively. These results provided novel insights into the roles of boron in resistance to CGMMV infection by regulating the expressions of ROS-related genes, laying a theoretical foundation for watermelon disease-resistance breeding.

## Materials and methods

### Plant growth and virus inoculation

*N. benthamiana* and watermelon (*Citrullus lanatus* cv “Jing Miguan 2”) plants were cultured in the artificial climate chamber (25°C day and night, 16 h light and 8 h dark cycles, 60% moisture). *N. benthamiana* leaves were sprayed twice every three days with H_2_O or H_3_BO_3_ with concentration of 100 mg·L^-1^ at 6-leaf stage before and after inoculation with phosphate-buffered saline (PBS solution) or CGMMV, respectively (PBS solution + H_2_O, P+H; PBS solution + boron, P+B; CGMMV + H_2_O, C+H; CGMMV + boron, C+B). The source of CGMMV (CGMMV-lnxg, GenBank ID: KY040049) was isolated and preserved on bottle gourd plants. The crude extracts were prepared from 0.1 g of CGMMV-infected bottle gourd leaves *via* homogenization with 0.01 mol·L^-1^ PBS (pH = 7.2) to mechanically inoculate the lower two leaves of *N. benthamiana* at the third day after spraying H_3_BO_3_ solution. At 9 days post inoculation (dpi), the upper two leaves were harvested for measurement of viral accumulations and RNA-seq analysis. Each treatment (P+H, P+B, C+H, C+B) was performed for three biological replicates with at least nine plants.

### RNA-seq analyses

The upper two leaves collected from at least five *N. benthamiana* plants were pooled for RNA-seq with three biological replicates in P+H, P+B, C+H and C+B. Total RNA was extracted from the pooled samples using a TIANGEN Total RNA Extraction Kit (Tiangen, Beijing, China). About 1 μg of total RNA from each sample was used as input for RNA preparation. Sequencing libraries were generated using a NEBNext Ultra RNA Library Prep Kit (New England Biolabs, Ipswich, USA). Briefly, mRNA was purified using poly-T oligo-attached magnetic beads. After fragmentation, cDNAs were synthesized and purified by AMPure XP system (Beckman Coulter, Beverly, USA). Then the purified double-strand cDNAs were washed for end repair and ligated to adapters. Finally, cDNA libraries were obtained by PCR enrichment and sequenced through the Illumina NovaSeq 6000 sequencing platform (Biomarker Biology Technology Co. Ltd., Beijing, China). The sequencing data were deposited in the SRA database at NCBI with the accession number PRJNA749605.

The clean reads were mapped to the reference genome of *N. benthamiana* (https://solgenomics.net/organism/Nicotiana_benthamiana/genome) using HISAT2 software. The relative gene expression levels were normalized as fragments per kilobase of transcript per million mapped reads (FPKM). The differentially expressed genes (DEGs) were selected through the threshold of false discovery rate (FDR) < 0.05 and |log2 fold change| ≥ 1. Seven databases, including Nr, Nt, KO, GO, Pfam, Swiss-Prot and KOG/COG, were used to annotate gene function. GO enrichment of DEGs were analyzed by a GOseq R packages based Wallenius non-central hyper-geometric distribution (Young et al., 2010). KEGG pathway enrichment analyses of DEGs were performed by KOBAS software (Mao et al., 2005).

### Co-expression network analyses

Weighted gene co-expression network analysis (WGCNA) was used to construct gene co-expression networks. Highly co-expressed gene modules were obtained using the WGCNA v3.1.1 package in R language (Langfelder and Horvath, 2008). A gene expression adjacency matrix was constructed to analyze the network topology with an unsigned type of topological overlap matrix (TOM), a power β of 5, a minModuleSize of 30, and a mergeCutHeight value of 0.25.

### Virus-induced gene silencing (VIGS) assays

In this study, nine genes were silenced using a tobacco rattle virus (TRV)-based VIGS vector in *N. benthamiana*. The fragments were cloned from *N. benthamiana* cDNA using specific primers with homologous arms (**Supplementary Table 1**) and PrimeSTAR^®^ Max DNA Polymerase (TaKaRa, Dalian, China) through PCR. Then, these PCR fragments were ligated into a pTRV2 vector that was digested using *BamH* I and *Xho* I restriction enzymes. These recombinant pTRV2 plasmids and pTRV1 plasmid were transformed into *Agrobacterium tumefaciens* strain GV3101, respectively. After propagation, the bacteria were re-suspended by infiltration buffer (10 mM MES, 10 mM MgCl_2_, 150 μM AS). Finally, the cultures of pTRV1 and recombinant pTRV2 were diluted to OD600 = 1.0, and mixed in equal volume. The lower two leaves of *N. benthamiana* at the 8-10 leaf stage were infiltrated with these cultures. A TRV-NbPDS containing a 409-bp fragment of *NbPDS* gene was used as a control. The upper two non-infiltrated *N. benthamiana* leaves were mechanically inoculated with CGMMV or PBS solution after 10 days post infiltration with TRV. At 10 dpi with CGMMV, the upper two leaves were collected for measure gene silencing efficiency and CGMMV accumulations.

Four watermelon genes were silenced by a pV190 vector, respectively. These fragments were cloned from watermelon cDNA using specific primers (**Supplementary Table 1**) through PCR as above described. Then, these fragments were ligated to the pV190 vector that was digested using *BamH* I restriction enzyme. These recombinant pV190 plasmids were transformed into *Agrobacterium tumefaciens* strain GV3101, respectively. After propagation, the bacteria were re-suspended by infiltration buffer (10 mM MES, 10 mM MgCl_2_, 100 μM AS) and diluted to the final OD_600_ = 1.0 as previous reports (Liu et al., 2020; Bi et al., 2022). The fully expanded cotyledons of watermelon at the 12-leaf stage were infiltrated with these cultures. A pV-ClPDS vector containing a 300-bp fragment of *ClPDS* gene was used as a control. After 32 days, the upper fourth watermelon tissue leaves were collected for measure gene silencing efficiency and CGMMV accumulations.

### Real-time quantitative reverse transcription-PCR (RT-qPCR)

The upper two *N. benthamiana* leaves and the upper fourth watermelon tissue leaves in VIGS assays, and the upper two leaves used for RNA-seq analyses were collected to perform RT-qPCR. Total RNA was extracted from plant leaves by TRIzol reagent (Tiangen, Beijing, China) and was synthesized to cDNA by HiScrit III RT SuperMix (+gDNA wiper) (Vazyme, Nanjing, China). ChamQ SYBR qPCR Master Mix (Vazyme, Nanjing, China) and StepOnePlus™ Real-Time PCR System (Applied Biosystems, Foster, USA) were used for RT-qPCR. *N. benthamiana Actin* gene (AY179605.1) and watermelon *Actin* gene (Cla007792) were used as an internal control, respectively. The specific primers for qPCR detection were designed according to gene sequences of *N. benthamiana* and watermelon (**Supplementary Table 1**). Relative expression levels of these genes were calculated using the 2^-ΔΔCT^ method (Schefe et al., 2006). RT-qPCR assays were performed with three biological replicates.

### Western blotting

Total protein was extracted from the same samples for RT-qPCR assays using a Plant Protein Extraction Kit (KeyGEN BioTECH, Jiangsu, China). Total protein was separated by 12% SDS-PAGE electrophoresis and transferred to 0.2 μm polyvinylidene fluoride (PVDF) membranes (Sangon Biotech, Shanghai, China). The sample proteins were detected by immunoblotting with anti-CGMMV monoclonal antibodies, followed by anti-mouse IgG horseradish peroxidase (HRP) antibody (Solarbio, Beijing, China). The membranes were placed into ECL solution (Millipore, Billerica, USA) to detect signals by Tanon Chemiluminescence Gel Imager (Tanon, Shanghai, China).

### Statistical analyses

All the data were analyzed using IBM SPSS Statistics 25 software (IBM Inc., Armonk, USA). The differences among groups were analyzed through two-tailed *t* test and one-way analysis of variance (Duncan).

## Results

### Foliar application of boron alleviated CGMMV infection in *N. benthamiana*

**Figure 1:**
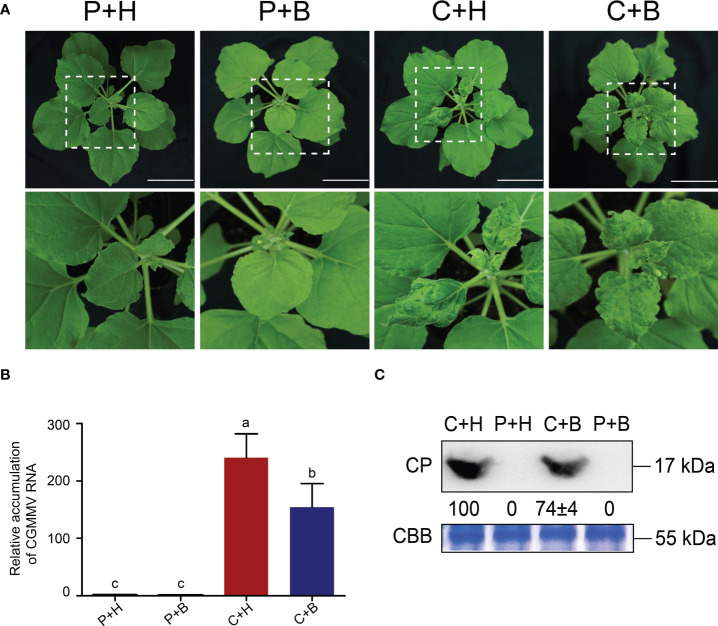
Symptoms and viral accumulation after CGMMV infection in *Nicotiana benthamiana* under different treatments. **(A)** Symptoms of *N. benthamiana* in four groups at 9 dpi. Scale bar = 5.0 cm. **(B)** The accumulation of CGMMV RNAs in upper leaves of *N. benthamiana* determined by RT-qPCR. Lowercase letters indicate statistical difference between treatments. The statistical significances were determined using one-way analysis of variance followed by Duncan’s multiple comparison test (*P* value < 0.05). **(C)** The accumulation of CGMMV CP in upper leaves of *N. benthamiana* determined by Western blotting. Coomassie brilliant blue stained cellular proteins were used as loading controls.

To verify the effects of boron on CGMMV infection in *N. benthamiana*, four treatments (PBS solution + H_2_O, P+H; PBS solution + boron, P+B; CGMMV + H_2_O, C+H; CGMMV + boron, C+B) were performed. The results revealed that the leaves of C+H-treated plants turned distorted and mosaic, while C+B-treated plants showed only slight mosaic and mottling at 9 dpi (**Figure 1A**). Meanwhile, P+H- and P+B-treated plants grew normally. Based on RT-qPCR and Western blot analyses, the accumulations of CGMMV genomic RNAs and CP proteins in C+B plants were decreased by 35% and 25%, respectively, compared with that in C+H plants (**Figures 1B, C**). These results demonstrated that foliar application of boron could alleviate CGMMV infection in *N. benthamiana*.

### RNA-seq analyses

**Figure 2:**
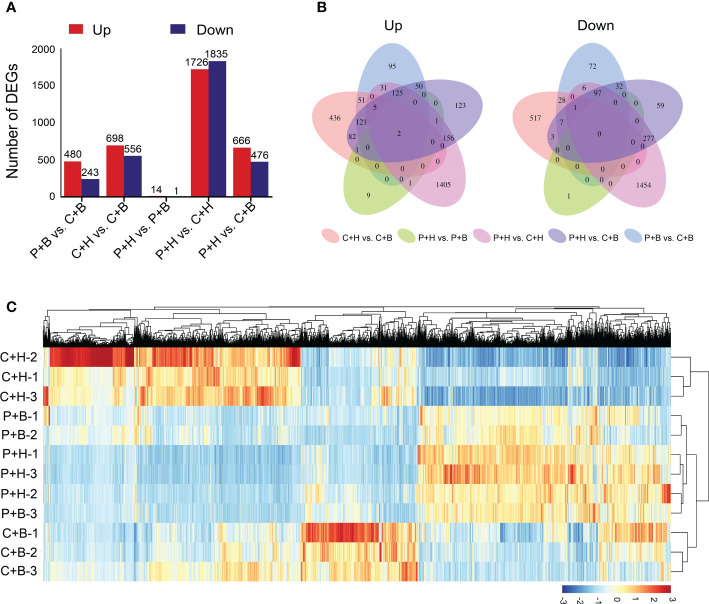
Statistical analyses of differentially expressed genes (DEGs). **(A)** The number of DEGs in five comparisons. **(B)** Venn diagrams showed the relationship of DEGs among five comparisons. The first venn diagram represents all up-regulated DEGs, and the second venn diagram represents all down-regulated DEGs. **(C)** The heatmap of hierarchical clustering of gene expression profiles in these four treatments with three biological replicates. The color scale (blue to red) represents low to high gene expression intensities. P+H, PBS solution + H_2_O; P+B, PBS solution + boron; C+H, CGMMV + H_2_O; C+B, CGMMV + boron.

To further clarify the roles of boron in alleviating CGMMV infection, RNA-seq was performed using the samples from P+H, P+B, C+H and C+B *N. benthamiana* plants. A total of 79.61 Gb of clean data were obtained from 12 libraries, of which each library contained ≥ 5.79 Gb of data with Q30 quality scores ≥ 93.54%, and CG content percentage between 43.43% and 44.13% (**Supplementary Table 2**). Mapped the sequencing reads to reference genome of *N. benthamiana*, the comparison efficiency ranged from 94.38% to 95.70% (**Supplementary Table 3**). Based on fold change ≥ 2 and FDR < 0.05 as the standard, a total of 4,570 DEGs were identified, of which 723, 1254, 15, 3561, and 1142 DEGs were found in P+B vs. C+B, C+H vs. C+B, P+H vs. P+B, P+H vs. C+H, and P+H vs. C+B, respectively (**Figure 2A** and **Supplementary Table 4**). Compared with that in C+H and P+B libraries, 698 and 480 DEGs were up-regulated, while 556 and 243 DEGs were down-regulated in C+B library, respectively. Moreover, 289 and 375 DEGs were up- and down-regulated in both P+H vs. C+H and P+H vs. C+B, respectively (**Figure 2B**). The heatmap showed hierarchical clustering of gene expression profiles in these four treatments with three biological replicates (**Figure 2C**). In addition, the expression patterns of the DEGs in P+H plants were strongly correlated with that in P+B plants (**Supplementary Figure 1A**). Principal component analysis (PCA) revealed that the expression profiles of C+H was divergent from that of C+B, P+H and P+B (**Supplementary Figure 1B**). These results indicated that CGMMV infection resulted in a significant change in the overall level of transcriptome profile of *N. benthamiana*, while foliar application of boron alleviated these effects by regulating gene expression.

### Functional classification of DEGs by GO and KEGG pathway analyses

**Figure 3:**
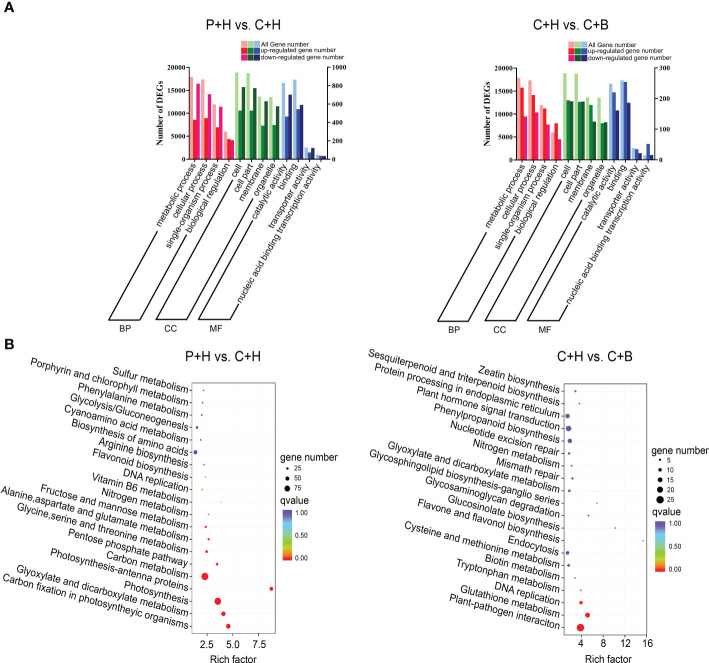
Enrichment analyses of DEGs. **(A)** GO analyses of DEGs in P+H vs. C+H and C+H vs. C+B in three main categories. The vertical axis on the left is all gene number. The vertical axis on the right is up- and down- regulated gene number. (MF, molecular function, CC, cellular component, BP, biological process). **(B)** KEGG pathway enrichment analyses of DEGs in P+H vs. C+H and C+H vs. C+B. P+H, PBS solution + H_2_O; C+H, CGMMV + H_2_O; C+B, CGMMV + boron.

GO terms and KEGG pathway enrichment were used to further analyze the functional classification of DEGs involved in the resistance of *N. benthamiana* to CGMMV infection regulated by boron. GO enrichment analyses revealed that in P+H vs. C+H, DEGs were mainly enriched in ‘single-organism process’, ‘metabolic process’ and ‘cellular process’ terms in biological process (BP) category, ‘cell’, ‘cell part’, ‘membrane’ and ‘organelle’ in cellular component (CC) category, ‘catalytic activity’ and ‘binding’ terms in molecular function (MF) category (**Figure 3A** and **Supplementary Table 5**). In C+H vs. C+B, DEGs were concentrated in the ‘metabolic process’ and ‘cellular process’ of BP, ‘cell’ and ‘cell part’ of CC, ‘binding’ and ‘catalytic activity’ of MF (**Figure 3A** and **Supplementary Table 5**). Interestingly, there were more down-regulated DEGs in most terms of GO enrichment in P+H vs. C+H, while more up-regulated DEGs in C+H vs. C+B (**Figure 3A** and **Supplementary Table 5**). To analyze whether the DEGs were significantly different in a certain pathway, a significant enrichment analysis was performed with the pathway in the KEGG database. The top 20 pathways with the smallest significant Q value were listed (**Figure 3B** and **Supplementary Table 6**). In P+H vs. C+H, DEGs were significantly enriched in ‘carbon metabolism’ (ko01200), ‘photosynthesis’ (ko00195), ‘glyoxylate and dicarboxylate metabolism’ (ko00630), ‘carbon fixation in photosynthetic organisms’ (ko00710), ‘pentose phosphate pathway’ (ko00030), ‘fructose and mannose metabolism’ (ko00051) and ‘nitrogen metabolism’ (ko00910). In C+H vs. C+B, DEGs were mainly enriched in the ‘plant-pathogen interaction’ (ko04626), ‘glutathione metabolism’ (ko00480), ‘DNA replication’ (ko0006270), ‘glycosaminoglycan degradation’ (ko00531), ‘tryptophan metabolism’ (ko00380) and ‘plant hormone signal transduction’ (ko04075). These results suggested that CGMMV infection affected carbohydrate metabolism and energy production in *N. benthamiana* leaves, while boron application could regulate the expressions of DEGs involved in signal transduction, antioxidation, replication, recombination and repair during CGMMV infection.

### Co-expression network analyses of DGEs by WGCNA

**Figure 4:**
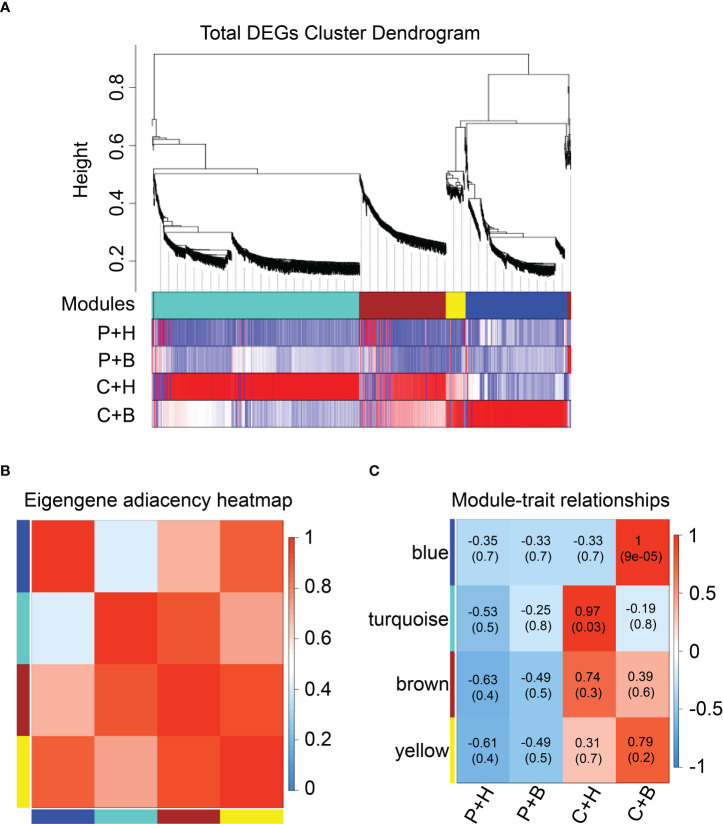
Weighted correlation network analysis (WGCNA) of DEGs. **(A)** Hierarchical cluster tree and heatmap of all DEGs. The hierarchical cluster tree shows co-expression modules identified through WGCNA. Each leaf in the tree represents one DEG. The major tree branches constitute four modules labelled with different colors. The heatmap shows the relative expressions of the whole DEGs in different modules. **(B)** Eigengene adjacency heatmap of the four modules shows the correlations among different modules. The darker red represents a higher correlation. **(C)** Associations between modules and traits. The colors of the modules are the same as that shown in **(a)** and **(b)**. The numbers in individual cells represent the correlations and the *P* values. P+H, PBS solution + H_2_O; P+B, PBS, PBS solution + boron; C+H, CGMMV + H_2_O; C+B, CGMMV + boron.

To clarify the gene regulatory network of boron against CGMMV infection in *N. benthamiana*, WGCNA was performed using all DEGs obtained. Through the clustering of DEGs, a total of four different color modules were obtained, including 81 DEGs in yellow module, 840 DEGs in turquoise module, 367 DEGs in brown module, and 415 DEGs in blue module (**Figure 4A** and **Supplementary Table 7**). Heat maps were used to show the correlation between modules and modules, modules and different treatments (**Figures 4B, C**). The results showed that blue and yellow modules were highly correlated, of which the DEGs were highly expressed in the C+B group (**Figures 4B, C**). These DEGs were mainly enriched in plant-pathogen interaction, protein processing in endoplasmic reticulum, glutathione metabolism and plant hormone signal transduction by Top GO and KEGG pathway analyses (**Supplementary Tables 8**, **9**). Moreover, these two modules mainly included DEGs related to ROS scavenging (*NbGST*, *NbPOD*), Ca^2+^ signal transduction (*NbCAM*/*NbCMLs*), hormone regulation (*NbERFs*) and plant immune-related TFs (*NbMYBs*, *NbWRKYs*, *NbNACs*) (**Supplementary Tables 8**, **9**). The turquoise and brown modules were highly correlated with C+H group. These DEGs were mainly enriched in DNA replication, phenylpropanoid biosynthesis, starch and sucrose metabolism, plant hormone signal transduction and carbon metabolism (**Supplementary Tables 8**, **9**), which were mainly involved in DNA binding (*NbDof*, *NbSPLs*, *NbHD-Zip*), ascorbic acid metabolism (*NbAO*, *NbGME*), flavonoid metabolism (*Nb4CL*, *NbCHS*), ROS scavenging (*NbCat*, *NbPOD*), and hydrolase activity (*NbGELPs*, *NbXTH*, *NbAEE*) (**Supplementary Tables 8**, **9**).

Through WGCNA analyses, we found that most of DEGs were related to plant signal transduction, secondary metabolism, hormone and immune-related TFs. Meanwhile, the DEGs in blue and yellow modules might play important roles in resistance of *N. benthamiana* to CGMMV infection regulated by boron.

### Analyses of DEGs in inositol phosphate metabolism

**Figure 5:**
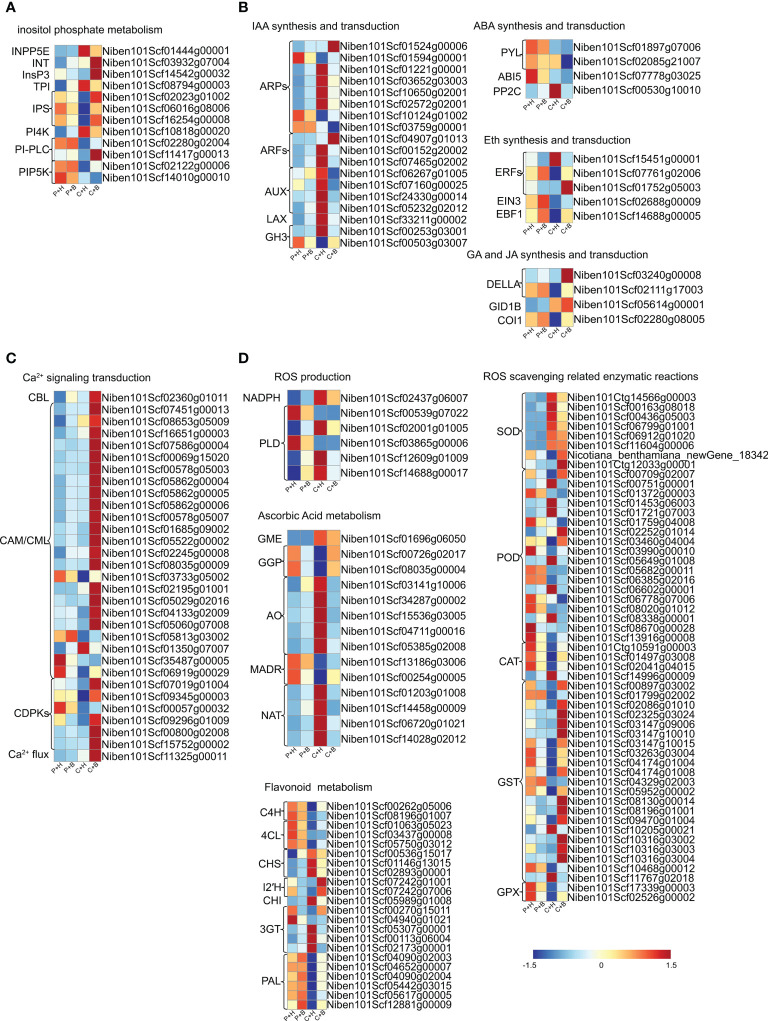
Heat map of the expression levels of DEGs involved in different pathways in *N. benthamiana*. **(A)** DEGs in inositol phosphate metabolism. **(B)** DEGs in phytohormones. **(C)** DEGs in Ca^2+^ signaling transduction. **(D)** DEGs in ROS metabolism. P+H, PBS solution + H_2_O; P+B, PBS, PBS solution + boron; C+H, CGMMV + H_2_O; C+B, CGMMV + boron.

It has been reported that the inositol phosphate metabolism pathway participates in response to biotic and abiotic responses in plants (Chaouch and Noctor, 2010). In this study, 12 DEGs involved in the inositol phosphate metabolism pathway were identified (**Figure 5A** and **Supplementary Table 10**). Six DEGs were down-regulated that were involved in inositol biosynthesis (*NbIPS*), phosphoinositol hydrolysis (*NbPI-PLC*) and phosphatidylinositol phosphorylation (*NbPIP5K7*, *NbPIP5K9*), while 3 DEGs related to phosphatidylinositol dephosphorylation (*NbINPP5E*) and phosphatidylinositol signal transduction (*NbPI4K*) were up-regulated in P+H vs. C+H. Interestingly, the accumulation of *NbIPS*, a key rate-limiting enzyme gene in inositol synthesis pathway, was induced by boron application in C+H vs. C+B. These results suggested that inositol biosynthesis, phosphoinositol hydrolysis and phosphatidylinositol phosphorylation might be inhibited by CGMMV infection, while inositol biosynthesis was promoted by boron under CGMMV infection.

### Analyses of DEGs in phytohormones

Plant normal physiological activities depend on the synergistic regulation of hormone networks, of which the roles in disease resistance have been extensively studied (Bhuvnesh et al., 2021). In auxin pathway, a total of 18 DEGs were identified (**Figure 5B** and **Supplementary Table 10**), including eight auxin-responsive proteins (*NbARPs*), three auxin-response factors (*NbARFs*), four auxin-induced proteins (*NbAUXs*), one auxin transporter-like protein (*NbLAX*) and two indole-3-acetic acid-amido synthetases (*NbGH3s*). The expression levels of most *NbARPs*, *NbARFs*, *NbLAX* and *NbGH3s* were changed in P+H vs. C+H. Among them, the expression levels of *NbARPs* were up-regulated, while *NbARFs* and *NbGH3s* were down-regulated in C+H vs. C+B, suggesting that these DEGs regulated by boron might be involved in resistance to CGMMV infection. Four DEGs related to abscisic acid (ABA) pathways were obtained (**Figure 5B** and **Supplementary Table 10**). In C+H vs. C+B, only phosphatase 2C51 (*NbPP2C51*) was down-regulated. In P+H vs. C+H, *NbPP2C51* remain unchanged, while abscisic acid receptor (*NbPYL4*) and abscisic acid-insensitive 5 (*NbABI5*) were down-regulated. In ethylene pathway, five DEGs, including three ethylene-responsive transcription factors (*NbERFs*), ethylene-insensitive 3 (*NbEIN3*) and EIN3-binding F-box protein 1 (*NbEBF1*) were identified (**Figure 5B** and **Supplementary Table 10**), of which four DEGs were obtained expect for *NbERF3* in P+H vs. C+H. *NbERF1B* and *NbERF3* were up-regulated in C+H vs. C+B. There were four DEGs related to gibberellic acid (GA) and jasmonic acid (JA) pathways (**Figure 5B** and **Supplementary Table 10**). In P+H vs. C+H, the accumulations of coronatine-insensitive protein 1 (*NbCOI1*) and DELLA protein RGL1 (*NbRGL1*) were deduced, while gibberellin receptor GID1B (*NbGID1B*) was induced. In C+H vs. C+B, only DELLA protein GAI (*NbGAI*) was significantly up-regulated. These results suggested that ABA catabolism and GA signal transduction might be inhibited, while ethylene synthesis was promoted by boron under CGMMV infection.

### Analyses of DEGs in Ca^2+^ signaling transduction

Ca^2+^ is the second messenger in plants, and its flow in and out of cells are induced by various biotic and abiotic stresses (Volk et al., 2004). In this study, 31 DEGs in Ca^2+^ signaling transduction were obtained (**Figure 5C** and **Supplementary Table 10**), of which 20 DEGs in C+H vs. C+B were up-regulated, such as *NbCAM*/*NbCMLs* and calcium-dependent protein kinase (*NbCDPKs*). In P+H vs. C+H, eight DEGs, including *NbCDPKs* (*NbCDPK28*, *NbCDPK29* and *NbCDPK32*) and *NbCAM*/*NbCMLs* (*NbCML1*, *NbCML3* and *NbCML21*), were down-regulated. These results suggested that Ca^2+^ signaling transduction might be promoted by boron under CGMMV infection.

### Analyses of DEGs in ROS metabolism

Plants use antioxidant systems to cope with ROS eruptions caused by stresses (Halliwell, 2006). Ascorbic acid metabolism and flavonoids metabolism are important components for non-enzymatic reaction of ROS scavenging. Enzymatic reaction mainly involved in CAT, GST and other antioxidant enzymes (Kadota et al., 2015). In this study, eight DEGs associated with ROS production were identified in P+H vs. C+H (**Figure 5D** and **Supplementary Table 10**), including a NADPH oxidase and seven phospholipase D (*NbPLD*). A total of 53 DEGs related to enzymatic reaction of ROS scavenging were identified (**Figure 5D** and **Supplementary Table 10**), including *NbGSTs*, *NbSODs*, *NbPODs*, *NbCATs* and *NbGPXs*. In C+H vs. C+B, most of *NbGSTs*, *NbCATs*, *NbGPXs* and several *NbPODs* were up-regulated, while other *NbPODs* were down-regulated. However, in P+H vs. C+H, most *NbGSTs*, *NbCATs*, *NbGPXs* and several *NbPODs* were down-regulated, while other *NbPODs* and most *NbSODs* were up-regulated. Moreover, in P+H vs. C+H, the expression levels of almost all genes involved in ascorbic acid metabolism and flavonoids metabolism were significantly changed in response to CGMMV infection (**Figure 5D** and **Supplementary Table 10**). In C+H vs. C+B, L-ascorbate oxidase (*NbAO*) and nucleobase-ascorbate transporter (*NbNAT*) were down-regulated, while phenylalanine ammonia-lyase (*NbPALs*) were up-regulated (**Figure 5D** and **Supplementary Table 10**).

### RT-qPCR validation of RNA-seq data

**Figure 6:**
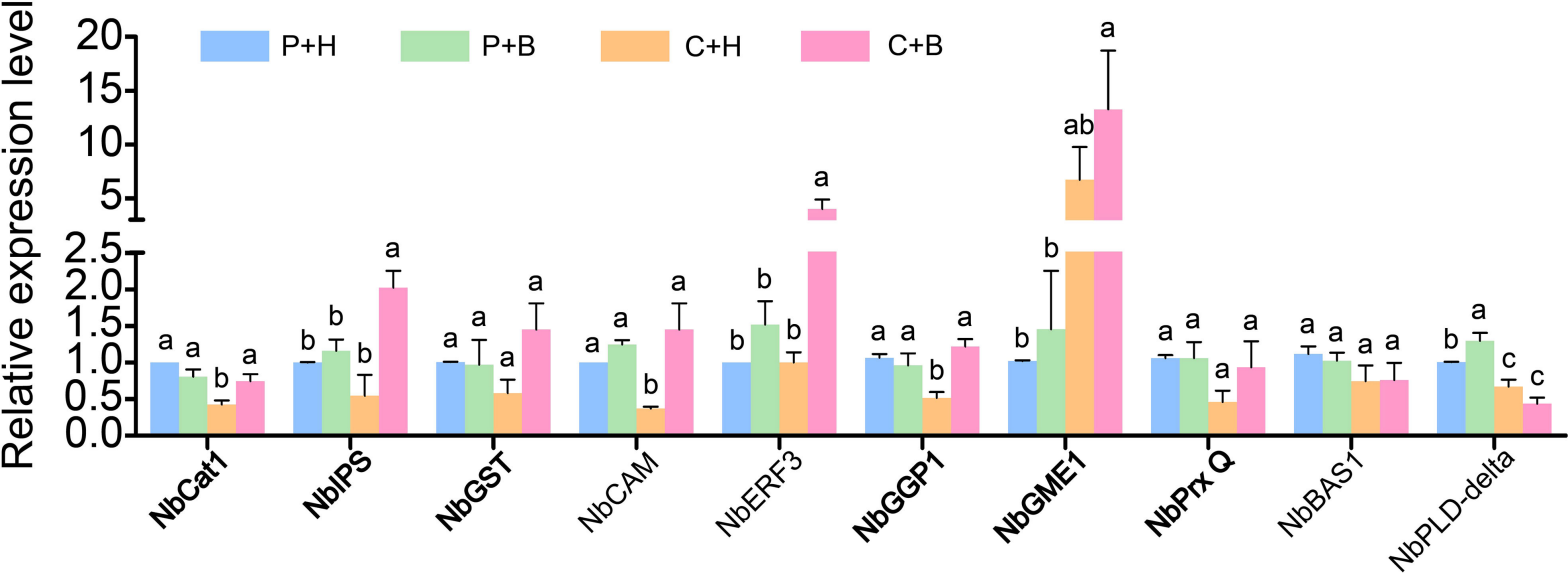
The expression levels of ten ROS-related genes were determined by RT-qPCR. Lowercase letters indicate statistical difference between treatments. The statistical significances were determined using one-way analysis of variance followed by Duncan’s multiple comparison test (*P* value < 0.05). P+H, PBS solution + H_2_O; P+B, PBS, PBS solution + boron; C+H, CGMMV + H_2_O; C+B, CGMMV + boron.

To confirm the accuracy of RNA-seq data, 10 DEGs associated with ROS metabolism were selected for RT-qPCR validation (**Figure 6**). The results showed that the expression levels of *NbCat1*(Niben101Ctg10591g00003), *NbPLD-delta* (Niben101Scf03865g00006), *NbCAM* (Niben101Scf01685g09002), *NbPrx Q* (Niben101Scf06385g02016), *NbIPS* (Niben101Scf16254g00008), *NbGGP1*(Niben101Scf00726g02017), *NbGST* (Niben101Scf00897g03002) and *NbBAS1* (Niben101Scf01372g00003) were down-regulated by CGMMV infection, while *NbGME1* (Niben101Scf01696g06050) was up-regulated. In C+H vs. C+B, the accumulations of *NbCat1*, *NbERF3* (Niben101Scf01752g05003), *NbCAM*, *NbPrx Q*, *NbIPS*, *NbGST* and *NbGGP1* were induced by boron application under CGMMV infection, while *NbGME*, *NbPLD-delta* and *NbBAS1* remained unchanged. These results were consistent with that of RNA-seq.

### Functional analyses of ROS-related genes in resistance to CGMMV infection in *N. benthamiana*

**Figure 7:**
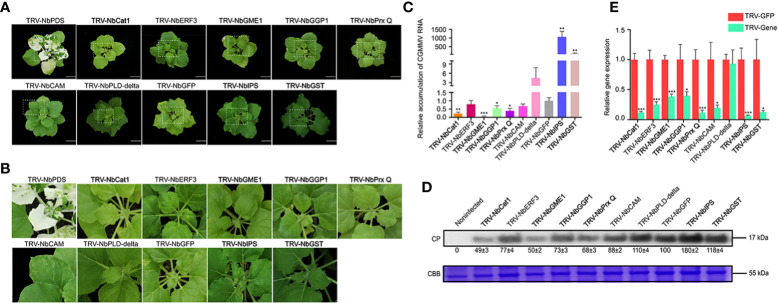
Functional analyses of nine ROS-related ggenes were determined to CGMMV infection through TRV-based VIGS assays in *N. benthamiana*. **(A)** Disease symptoms on different gene-silenced *N. benthamiana* plants after CGMMV infection. **(B)** Close-up views of upper leaves indicated by white dash boxes in **(A)**. **(C)** CGMMV accumulations determined by RT-qPCR in different gene-silenced *N. benthamiana*. Asterisks indicate statistical difference between treatments, determined by the two-tailed *t* test (*P < 0.05, **P < 0.01, ***P < 0.001). **(D)** The expression levels of CGMMV CP proteins in upper leaves of *N. benthamiana*. **(E)** Silencing efficiencies of target genes determined through RT-qPCR. Asterisks indicate statistical difference between treatments, determined by the two-tailed *t* test (*P < 0.05, **P < 0.01, ***P < 0.001).

It has been reported that ROS participates in stress responses induced by boron (Awan et al., 2019). In this study, nine ROS-related genes were selected to further explore their roles in resistance to CGMMV infection by TRV-based VIGS assays (**Figure 7**). After 10 days post CGMMV inoculation, the *NbCat1-*, *NbGME1-*, *NbGGP1-* and *NbPrx Q-*silenced plants showed mild symptoms, while silencing *NbGST* and *NbIPS* caused more severe symptoms than that in control groups, respectively. There was no significant difference in symptoms of *NbCAM*-, *NbERF3*- and *NbPLD-delta-*silenced plants (**Figures 7A, B**). The accumulations of CGMMV genomic RNAs and CP proteins were consistent with the symptom severity determined by RT-qPCR and Western blot assays, respectively (**Figures 7C, D**). The silencing efficiencies of these genes were between 60% and 90% detected by RT-qPCR, expect *NbPLD-delta* (**Figure 7E**).

### Functional analyses of ROS-related genes in watermelon resistance to CGMMV infection

**Figure 8:**
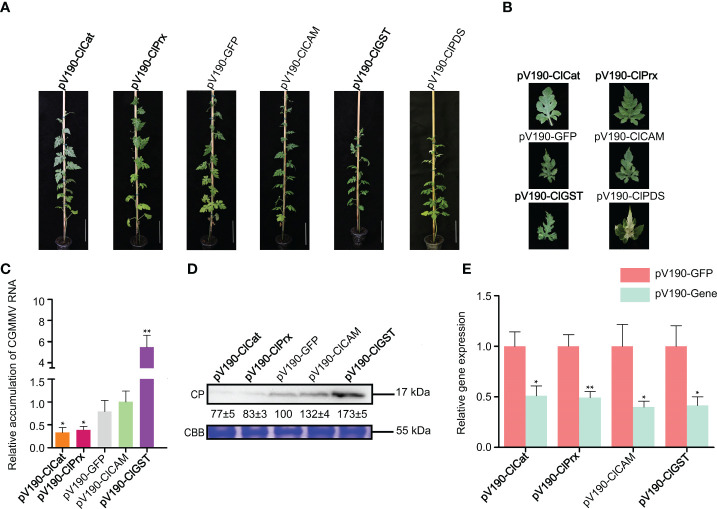
Functional analyses of four ROS-related genes through CGMMV-based VIGS assays in watermelon. **(A)** Disease symptoms on different gene-silenced watermelon plants after CGMMV infection. **(B)** Close-up views of the upper fourth leaves in watermelon. **(C)** CGMMV accumulations determined by RT-qPCR in different gene-silenced watermelon plants. Asterisks indicate statistical difference between treatments, determined by the two-tailed *t* test (*P < 0.05, **P < 0.01). **(D)** The expression levels of CGMMV CP proteins in the upper fourth leaves of watermelon. **(E)** Silencing efficiencies of target genes determined through RT-qPCR. Asterisks indicate statistical difference between treatments, determined by the two-tailed *t* test (*P < 0.05, **P < 0.01).

To further explore the roles of ROS-related genes in resistance to CGMMV infection in natural host plants, we selected four homologous genes to be silenced through CGMMV-based VIGS assays in watermelon plants (**Figure 8**). At 19 days post agroinfiltration (dpai), pV190-PDS-treated plants showed obvious bleaching on leaves, proving that pV190-based VIGS vector had successfully induced gene silencing in watermelon leaves. At 32 dpai, the *ClCat*- and *ClPrx-*silenced plants showed mild CGMMV symptoms, while *ClGST*-silenced plants showed more severe symptoms compared to pV190-GFP plants. The symptoms of *ClCAM*-silenced plants were similar to that of control groups (**Figures 8A,B**). Moreover, the accumulations of CGMMV genomic RNAs and CP proteins were decreased in *ClCat-* and *ClPrx-*silenced plants, while increased in *ClGST*-silenced plants (**Figures 8C,D**). The silencing efficiencies of target genes were 52~65% determined through RT-qPCR (**Figure 8E**).

## Discussion

In recent years, the roles of trace elements in regulating plant stress responses have been widely reported (Shireen et al., 2018; Riaz et al., 2020; Yin et al., 2021). Several studies have revealed the molecular mechanisms and regulatory networks of trace elements during plant antiviral defenses (González-Fontes and Fujiwara, 2020). In *Oryza sativa*, copper inhibits transcriptional activation of *MIR528* through regulating the protein level of SQUAMOSA promoter-binding-like protein 9 (SPL9), then alleviating miR528-mediated cleavage of *AO* transcripts to induce broad-spectrum virus resistance (Yao et al., 2022). In tobacco, zinc has a positive effect on inhibiting tobacco mosaic virus (TMV) infection by recruiting *ERF5* to promote inositol phosphate metabolism (Wang et al., 2022). Boron, one of the most important trace elements, maintains normal plant growth by stabilizing the structure of cell walls and regulating antioxidant systems (Shireen et al., 2021). Our previous data have demonstrated the effects of boron on watermelon resistance to CGMMV-induced watermelon blood flesh disease (WBFD) (Bi et al., 2022). In this study, we found that boron application could alleviate CGMMV infection in *N. benthamiana* plants (**Figures 1A–C**), which were consistent with the results obtained in our previous report (Bi et al., 2022).

Plants evolved a series of self-protection mechanisms during the continuous infection of pathogens (Xu et al., 2017). The natural immune system consists of immune responses induced by pathogen-associated pattern molecules (PAMP-triggered immunity, PTI) and an immune response activated by sensing effector proteins secreted by the pathogen (effector-triggered immunity, ETI) in plants (Jones and Dangl, 2006). The regulatory mechanisms of TFs in these processes have been widely reported (Wang et al., 2016). Moreover, TFs play an important role in plant growth and stress tolerance by interacting with cis-regulatory elements of target genes to regulate gene expression (Shiu et al., 2005). In our study, the expressions of 94 TFs were found to be regulated by boron in response to CGMMV infection, including WRKY, MYB, NAC, etc. These TFs are basically consistent with those reported by Sun et al. in watermelon leaves in response to CGMMV infection (Sun et al., 2019). WRKYs bind to a consensus cis-element referred to the “W-box” (TTGACT/C) in the promoter regions to regulate defense-related genes (Wani et al., 2021). Some studies have shown that WRKY TFs regulate many stress reactions in plants by affecting biosynthesis and signal transduction of plant hormones (Rushton et al., 2010). Previous studies have verified that many WRKY TFs can positively regulate SA pathway to mediate plant resistance to necrotrophic fungal and biotrophic bacterial pathogens (Zheng et al., 2006; Lai et al., 2008). On the contrary, some WRKY TFs have been verified to inhibit defense-related gene expressions, which is related to the inhibition of JA signaling (Brotman et al., 2013). In this study, 15 *NbWRKYs* (*NbWRKY40*, *NbWRKY6*, *NbWRKY11*, *NbWRKY31*, *NbWRKY53*, *NbWRKY41*) were found to be up-regulated in C+H vs. C+B (**Supplementary Table 10**). Therefore, we hypothesized that boron might promote SA signaling pathway by inducing the expression of some WRKY TFs to improve the resistance of *N. benthamiana* to CGMMV infection. It is well‐known that MYB TFs regulate the biosynthesis of plant secondary metabolites, including anthocyanidins and flavonoids, which play important roles in plant resistance to biotic and abiotic stresses (Ma et al., 2018). In Chinese wild grape (*Vitis davidii*), *VdMYB1* was reported to be involved in flavonoid metabolism by regulating the expression of stilbene synthase genes with function in resistance to powdery mildew (PM) disease (Yu et al., 2019). We found that boron spraying under CGMMV infection up-regulated the expression of five MYB TFs (*NbMYB44*, *NbMYB48*, *NbMYB57* and *NbMYB4*) and down-regulated the expression of *NbMYB86*, which may promote flavonoid metabolism to improve resistance to CGMMV infection (**Supplementary Table 10**). It has been reported that a variety of NAC TFs are involved in plant disease resistance (Kumar et al., 2021). Overexpression of *TaNAC1* in wheat inhibited SA signaling pathway and enhanced susceptibility to *Pseudomonas syringae* (Wang et al., 2015). Overexpression of *GhATAF1* in cotton can activate SA-mediated defense signaling and inhibit JA-mediated signal transduction, which enhances cotton susceptibility to *Verticillium dahlia* (He et al., 2016). In this study, we found that the expression levels of one *NbNAC14*, two *NbNAC2* and one *NbNAC68* were increased in C+H vs. C+B, while three *NbNAC8* and one *NbNAC73* were decreased (**Supplementary Table 10**). Therefore, we hypothesized that boron-induced resistance to CGMMV infection in *N. benthamiana* plants was related to JA and SA signaling pathways regulated by NAC TFs. In addition, we also found that some HD-Zip TFs in plants responded to CGMMV infection, but their accumulations were not induced by boron application (**Supplementary Table 10**).

Ca^2+^ is the second messenger of organisms (Volk et al., 2004), and CDPKs are important Ca^2+^ sensors (Sanyal et al., 2020). In response to plant stresses, the Ca^2+^ sensors convert calcium signals into physiological and biochemical reactions (Poovaiah et al., 2013). CDPKs-mediated signal transduction can be activated in PTI signaling pathways (Boudsocq et al., 2010). Overexpression of *AtCDPK1* induces SA accumulation to enhance the broad-spectrum resistance to pathogen infection in *Arabidopsis* (Coca and San, 2010). In this study, we found that the expression levels of *NbCDPK28*, *NbCDPK29* and *NbCDPK32* were down-regulated and *NbCDPK7* was unchanged in P+H vs. C+H. However, in C+H vs. C+B, *NbCDPK28* and *NbCDPK7* were up-regulated, while the expressions of *NbCDPK29* and *NbCDPK32* were unchanged (**Supplementary Table 4**). These results indicated that *NbCDPKs* regulated by boron application played important roles in resistance to CGMMV infection. The inositol phosphate metabolism system affects ROS production by regulating plant hormone and Ca^2+^ signaling (Gillaspy, 2013; Jia et al., 2019). Inositol is the core of inositol phosphate metabolism, and IPS is the first enzyme that catalyzes inositol synthesis (Kusuda et al., 2014). The expression of *IPS* gene was positively correlated with inositol content, which can improve the adaptability of plants to abiotic stresses (Das-Chatterjee et al., 2006). Here, the expression of *NbIPS* was up-regulated in C+H vs. C+B, and silencing of *NbIPS* expression increased CGMMV accumulation (**Figures 7**). These results suggested that *NbIPS* was a positive regulator of *N. benthamiana* resistance to CGMMV infection, of which the expression level was regulated by boron application.

Ethylene, one of the important plant hormones, regulates plant development and stress responses through a variety of signaling pathways (Binder, 2020). Studies have shown that ERFs induced the production of ROS by regulating the expressions of *respiratory burst oxidase* (Rboh) genes (Yao et al., 2017; Riester et al., 2019). ROS acted as signal molecules to transfer stress signals into cells, thereby activating ethylene signals (Wang et al., 2013). In this study, we found that the expression levels of *NbERF1B* and *NbERF3* were up-regulated by boron application in response to CGMMV infection, while silencing of *NbERF3* expression had no effect on CGMMV infection. Previous studies have shown that ethylene is not the key for plant resistance against viruses (Geri et al., 2004). Therefore, we hypothesized boron application induced the expressions of ERFs and promoted *N. benthamiana* resistance to CGMMV infection, which was related to ROS burst. However, *NbERF3* is not a key gene in this pathway for plant resistance to CGMMV infection.

**Figure 9:**
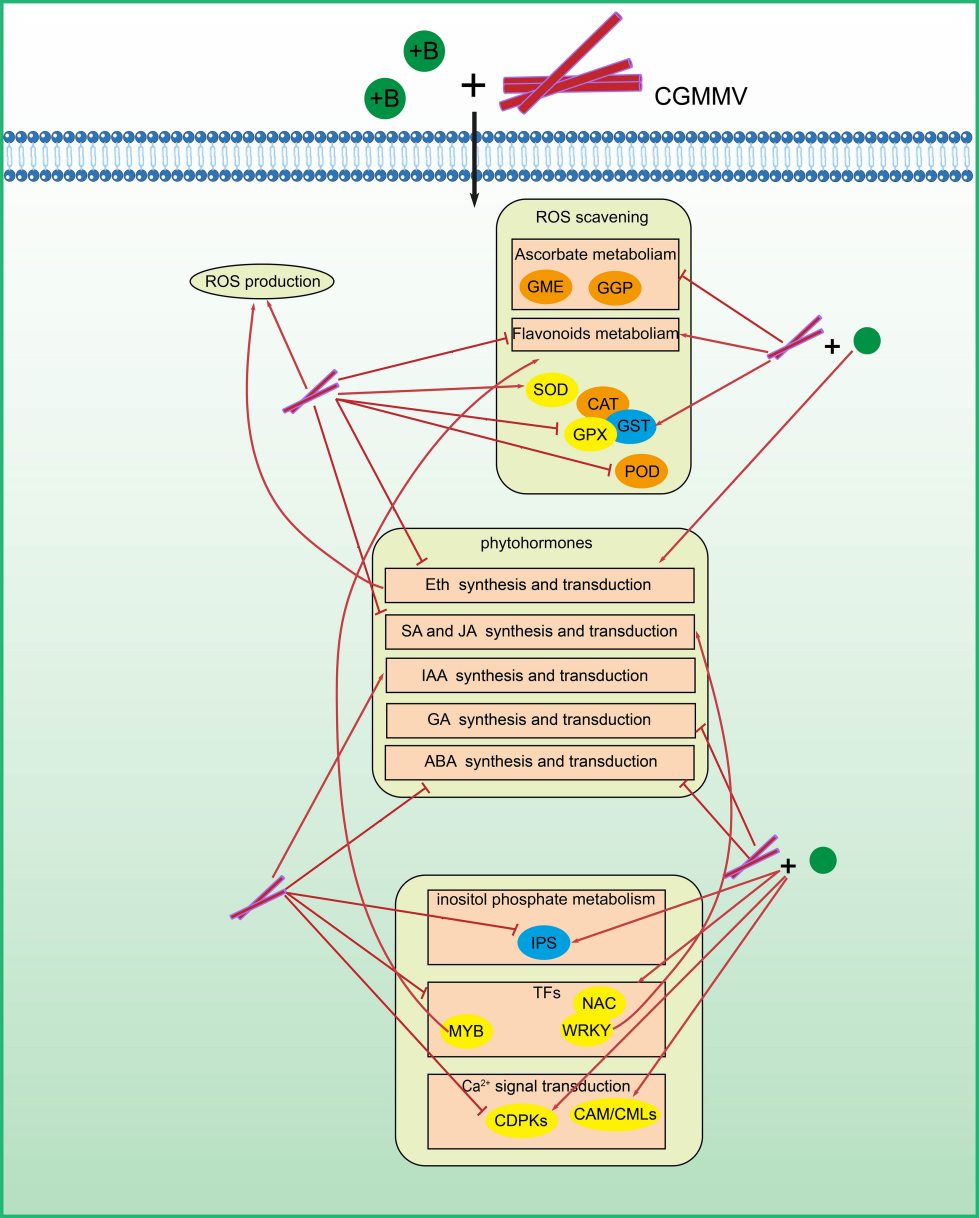
A proposed model for the possible roles of ROS-related genes in resistance to CGMMV infection regulated by boron. Genes in orange boxes were required for CGMMV infection in *N. benthamiana*. Genes in blue boxes played anti-CGMMV roles in *N. benthamiana*. Genes in yellow boxes responded to CGMMV infection and / or boron application.

ROS burst is one of the most important layers of plant defense responses against pathogen infection (Sewelam et al., 2016). Plant antioxidant systems consist of many enzymes, such as CAT, Prx and GST (Kadota et al., 2015). They regulate ROS homeostasis to avoid oxidative damage, and participate in signal transduction (Bolwell et al., 2002). Flavonoid metabolism and ascorbic acid metabolism are important secondary metabolic pathways in plants, which play an important role in plant antioxidant (Smirnoff, 2018; Shen et al., 2022). Based on the results of RNA-seq analyses, we found that the expression levels of 79 genes related to ROS scavenging were changed under CGMMV infection, of which 46 genes and other nine genes were regulated by boron application under CGMMV infection. These DEGs were involved in ascorbic acid metabolism (*NbGME*, *NbGGP*, *NbAO*, *NbMDAR* and *NbNAT*), flavonoid metabolism (*NbC4H*, *NbPAL*, *Nb4CL*, *NbCHS*, *Nb3GT* and *NbI2’H*) and ROS scavenging enzymes (*NbGSTs*, *NbSODs*, *NbCATs*, *NbPODs* and *NbGPXs*). RNA-seq and RT-qPCR analyses showed that the expressions of *NbCat1* and *NbGST* were up-regulated in C+H vs. C+B, while the expressions of *NbGGP*, *NbPrx Q* and *NbGME* were changed but not significantly. In P+H vs. C+H, *NbGGP*, *NbPrx Q*, *NbCat1* and *NbGST* were down-regulated, while *NbGME* was up-regulated. These results suggested that ROS-related genes regulated by boron played important roles in defense responses of *N. benthamiana* to CGMMV infection (**Figure 9**). *CATs*, as SA receptors, have been proved to associate with various virus infections (Yang et al., 2020; Jiao et al., 2021). The interactions between catalase and viral proteins affects the function of catalase (Murota et al., 2017). Silencing *CAT1* inhibited pepino mosaic virus (PepMV) accumulation due to PepMV TGBp1 (p26)-CAT1 interaction in *N. benthamiana*, which enhanced CAT activity and disrupted ROS homeostasis (Mathioudakis et al., 2013). GST is involved in redox reactions and flavonoid metabolism (Edwards et al., 2000; Shao et al., 2021). GST and GSH play synergistic roles in induction of a hypersensitive response (HR) in plant defense responses (Király et al., 2012). It was reported that overexpression of *GaGSTF9* in *Arabidopsis* significantly reduces the accumulation of *Verticillium dahliae* (Gong et al., 2018). In addition, the expression level of *GST* gene was negatively correlated with ROS content (Bibi et al., 2013). Prx is also an important regulator of hydrogen peroxide signaling with molecular chaperone function (Wood et al., 2003). In *PrxIIB*-silenced *N. benthamiana*, CAT activity and ascorbate (AsA) content continued to increase, which maintained the levels of H_2_O_2_ unchanged (Vidigal et al., 2015). AsA is widely reported in plants and assists plant cells in resisting oxidative stress (Smirnoff and Wheeler, 2000). GME and GGP are key enzymes in several AsA synthesis pathways, and the *GME*-silenced tomato plants had less AsA content (Gilbert et al., 2009). SlHZ24, a HD-Zip I family transcription factor in tomato, binds to the promoter of *GMP3*, and overexpression of SlHZ24 affected the expression of *GME3*, *GME1* and *GGP1* to regulate AsA metabolism (Hu et al., 2016). In this study, we silenced nine DEGs related to ROS metabolism to verify their roles in resistance to CGMMV infection using a TRV-based VIGS vector in *N. benthamiana* (**Figures 7**). The results indicated that *NbCat1*, *NbGME1*, *NbGGP1* and *NbPrx Q* were required for CGMMV infection, while *NbGST* played important roles in resistance to CGMMV infection. Then, we selected four genes to further validate their antiviral roles in watermelon by a CGMMV-based VIGS vector (**Figures 8**). The results revealed that *ClCat* and *ClPrx* were beneficial to CGMMV infection, while *ClGST* was hostile, which were consistent to the results obtained in *N. benthamiana*. In general, decreased ROS content inhibits downstream defense-related responses, and favors pathogen infection, while some pathogens induce ROS accumulation to accelerate cell oxidative damage to promote subsequent infection (Govrin and Levine, 2000; Mittler et al., 2022). After virus infection, the changes of enzyme activities in ROS scavenging were different (Clarke et al., 2002). We hypothesized that the enhanced anti-CGMMV roles induced by silencing *NbCat1*, *NbGME1*, *NbGGP1* and *NbPrx Q* might be associated with increased levels of ROS. However, *NbGST* plays a major role in maintaining ROS homeostasis, and silencing *NbGST* will lead to excessive accumulation of ROS, which might disrupt the anti-CGMMV responses of host plants. Moreover, except for the change in ROS contents, the changes in downstream defense-related genes that are affected by ROS signaling should also be considered. The specific mechanism needs to be further explored.

## Data availability statement

The datasets presented in this study can be found in online repositories. The names of the repository/repositories and accession number(s) can be found below: https://www.ncbi.nlm.nih.gov/, PRJNA749605.

## Author contributions

ZX and YW conceived the research project; XB completed transcriptome sequencing tests; HG performed transcriptome data analyses, gene function validation and RT-qPCR experiments; DJ and MC assisted in the completion of CGMMV-based VIGS assays. HG wrote the original draft; XB, ZW and MA offered research suggestions and revised the manuscript; ZX edited the final manuscript. All authors reviewed and approved the manuscript.

## Funding

This work was supported by grant from the Joint Fund for Innovation Enhancement of Liaoning Province (2021-NLTS-11-04), the National Key Research and Development Program of China (2018YFD0201300) and the Major Science and Technology R&D Project of Shenyang Municipal Government (Grant No. 17-146-3-00).

## Acknowledgments

We thank Prof. Qinsheng Gu (Zhengzhou Fruit Research Institute, Chinese Academy of Agricultural Sciences) for providing the pV190 vector, Prof. Yule Liu (School of Life Sciences, Tsinghua University, Beijing, China) for providing the pTRV1 and pTRV2 vectors, and Prof. Jianxiang Wu (Zhejiang University, Hangzhou, China) for providing CGMMV anti-CP antiserum.

## Conflict of interest

The authors declare that the research was conducted in the absence of any commercial or financial relationships that could be construed as a potential conflict of interest.

## Publisher’s note

All claims expressed in this article are solely those of the authors and do not necessarily represent those of their affiliated organizations, or those of the publisher, the editors and the reviewers. Any product that may be evaluated in this article, or claim that may be made by its manufacturer, is not guaranteed or endorsed by the publisher.
