# Supplementary material for: Reactive oxygen species-related genes participate in resistance to cucumber green mottle mosaic virus infection regulated by boron in Nicotiana benthamiana and watermelon
Source: Front Plant Sci. 2022 Nov 11;13:1027404. doi: 10.3389/fpls.2022.1027404 (PMC9691971; doi:10.3389/fpls.2022.1027404)
Supplement: Supplementary file 1 [file DataSheet_1.docx]

Supplementary Material

## Supplementary Figures


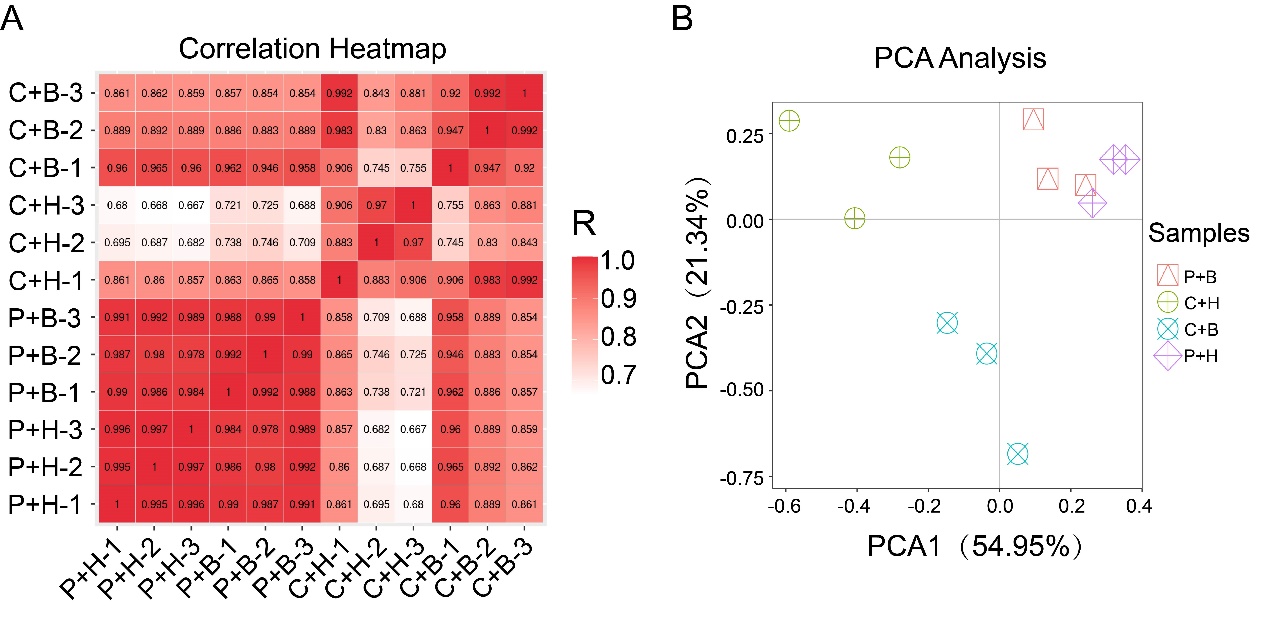


**Supplemental Figure 1.** **The correlation and PCA analyses of four treatments in *Nicotiana benthamiana***. **(A)** Correlation heatmap of four treatments with three biological replicates. The closer Pearson's Correlation Coefficient (R) is to 1, the stronger the correlation between the two duplicate samples. **(B)** Principal component analysis (PCA) of four treatments with three biological replicates. Scatter plot of PCA1 (x axis) versus PCA2 (y axis).
